# RESILIENCE OF CHINESE OLDER COUPLES LIVING WITH CHRONIC ILLNESSES

**DOI:** 10.1093/geroni/igz038.3544

**Published:** 2019-11-08

**Authors:** Esther O Chow

**Affiliations:** City University of Hong Kong, Hong Kong, China, Hong Kong

## Abstract

Applying theories of lifelong psycho-social growth and life course principles of human development, older couples generally possess rich and valuable resources from diverse aspects of life. However, empirical evidence, on whether these supports are fully transferred to benefit themselves as they age, remains uncertain due to inevitable physiological decline and increasing caregiving strains. The present study explicated the underlying processes of the older Chinese couples in forming a dyadic resilience model as they age with chronic illnesses. Using in depth interviews, 15 pairs of older Chinese couples were interviewed twice through home visits. The conversations were transcribed and analyzed using thematic analysis. Eight themes related to resilience and challenges of spousal caregiving were identified: 1) insights and acceptance of the chronicity of illnesses, 2) a sense of purpose and commitment as being the spouse in providing care for each other, 3) mastering skills and resources to handle the duties, 4) mixed feelings when the spouse was functioning changes, 5) realizing the importance of self-care and life-management, 6) cultivating positive meanings amidst difficult circumstances, 7) accepting things that cannot change, and cherishing on those aspects which are still functioning well, 8) working closely with other formal and informal support for common good. Findings have both practice and policy implications to health and social care practitioners in understanding different patterns of resilience as experienced by older Chinese couples in mitigating the developmental challenges from individual, relational and societal levels, and research implications in examining actor-partner interdependence practice in addressing caregiving challenges.

